# A multicentre prospective single‐arm clinical trial to evaluate the treatment outcomes of prophylactic laparoscopic lateral pelvic lymph node dissection for advanced lower rectal cancer

**DOI:** 10.1111/codi.70078

**Published:** 2025-04-01

**Authors:** Atsushi Hamabe, Junichi Nishimura, Yozo Suzuki, Masayoshi Yasui, Masakazu Ikenaga, Tsukasa Tanida, Shinichi Yoshioka, Yoshihito Ide, Yusuke Takahashi, Hiroshi Takeyama, Takayuki Ogino, Hidekazu Takahashi, Norikatsu Miyoshi, Makoto Fujii, Yuko Ohno, Hirofumi Yamamoto, Kohei Murata, Mamoru Uemura, Yuichiro Doki, Hidetoshi Eguchi

**Affiliations:** ^1^ Department of Gastroenterological Surgery Osaka University Osaka Japan; ^2^ Department of Gastroenterological Surgery Osaka International Cancer Institute Osaka Japan; ^3^ Department of Surgery Toyonaka Municipal Hospital Osaka Japan; ^4^ Department of Surgery Higashiosaka City Medical Center Osaka Japan; ^5^ Department of Surgery Yao Municipal Hospital Osaka Japan; ^6^ Department of Surgery Japan Community Health Care Organization Osaka Hospital Osaka Japan; ^7^ Department of Colorectal Surgery NHO Osaka National Hospital Osaka Japan; ^8^ Department of Surgery Suita Municipal Hospital Osaka Japan; ^9^ Department of Surgery Osaka Police Hospital Osaka Japan; ^10^ Division of Health and Sciences Osaka University Graduate School of Medicine Osaka Japan; ^11^ Department of Surgery Kansai Rosai Hospital Osaka Japan

**Keywords:** laparoscopic surgery, lateral pelvic lymph node dissection, rectal cancer, short‐term outcomes

## Abstract

**Aim:**

There has been no prospective multicentre validation of the treatment outcomes of minimally invasive lateral pelvic lymph node dissection for lower rectal cancer; hence, this prospective study aimed to evaluate the treatment outcomes of prophylactic laparoscopic lateral pelvic lymph node dissection.

**Method:**

Between May 2018 and August 2021, 90 patients with Stage II–III rectal cancer were registered. The clearance range for lateral pelvic lymph node dissection included the lymph nodes around the internal iliac artery and the obturator lymph nodes, while the autonomic nerves were generally preserved. The primary outcome was the incidence of Grade III–IV postoperative complications at discharge. The secondary outcomes were surgical and pathological outcomes, urinary function, sexual function, disease‐free survival and overall survival. The experience of each facility and surgeon requirements were set to maintain quality control of lateral pelvic lymph node dissection.

**Results:**

Of the 90 patients, 87 were analysed after exclusion of ineligible patients. There were 30 and 57 cases, respectively, of Stage II and III rectal cancer, among which 17 patients underwent neoadjuvant chemotherapy. The median operating time and blood loss were 472 min and 55 mL, respectively. Postoperative complications were observed in 22 patients (25.3%), and the primary outcome of Grade III postoperative complication was observed in five patients (5.7%). Eight lateral lymph nodes were harvested bilaterally, and lateral lymph node metastasis was observed in 14 patients.

**Conclusion:**

Prophylactic lateral pelvic lymph node dissection can be safely performed with adequately quality‐controlled laparoscopic procedures.

**What does this paper add to the literature?**

The current study demonstrated that lateral pelvic lymph node dissection for rectal cancer can be safely performed with adequately quality‐controlled laparoscopic procedures.

## INTRODUCTION

Treatment strategies for advanced rectal cancer have differed significantly between Western countries and Japan [1]. In Western countries, conventional treatment involves total mesorectal excision (TME) following preoperative chemoradiotherapy. In recent years, there has been a trend towards adopting total neoadjuvant therapy to achieve better control of distant metastases [2, 3, 4, 5]. In Japan, upfront surgery involving TME with lateral pelvic lymph node dissection (LLND) is the standard approach. LLND does not show efficacy in disease‐free survival, but exhibits effectiveness in local control, especially within the lateral compartments [6, 7, 8]. Recent reports have demonstrated the therapeutic benefits of LLND, even in patients who underwent preoperative treatments, such as chemoradiotherapy or total neoadjuvant therapy, and subsequently showed positivity for lateral lymph node metastasis [9, 10, 11]. It is anticipated that treatment strategies that combine preoperative therapy with LLND will continue to advance.

Although LLND, a technically demanding procedure, has traditionally been performed using open surgery, the increasing popularity of minimally invasive surgery (MIS) has led to its recent application in LLND. Retrospective reports from advanced facilities have indicated favourable outcomes regarding the safety and curative potential of MIS‐LLND [12, 13, 14, 15]. However, no prospective multicentre study has validated the treatment outcomes of prophylactic LLND performed using MIS, and the significance of the procedure remains unclear. This study aimed to prospectively evaluate the treatment outcomes of prophylactic laparoscopic LLND.

## METHODS

### Study population

This was a multicentre prospective observational study conducted at six facilities affiliated with the Multicentre Clinical Study Group of Osaka, Colorectal Cancer Treatment Group. The inclusion criteria were as follows: patients diagnosed with adenocarcinoma of the rectum via endoscopic biopsy of the rectal primary lesion, with the lower edge of the tumour located between the peritoneal reflection and the anal verge, cStage II or III (evaluation based on pretreatment findings for patients who underwent preoperative treatment), absence of lateral lymph node metastasis with a short‐axis diameter of ≥10 mm on CT and MRI, aged between 20 and 80 years, and scheduled for laparoscopic LLND. The exclusion criteria were active multiple cancers and lack of decision‐making capacity. Similar to the JCOG0212 trial [16], we set the upper limit for the lymph node size to a short axis of 10 mm and excluded patients requiring LLND for therapeutic intent involving obvious lateral lymph node metastasis. Regarding preoperative treatment, only patients who had undergone neoadjuvant chemotherapy, which was commonly administered within the research group at the time, were eligible for registration. Patients receiving radiotherapy were excluded because they were considered a non‐standard treatment at the time and raised concerns about increased surgical risks. Cases were registered before surgery, and patients who received preoperative treatment were registered after chemotherapy. The study protocol was approved by the ethics committees of the participating institutions and consent forms were obtained from all participants. This study was registered with the University Hospital Medical Information Network Clinical Trials Registry (identification number UMIN000033355).

### Surgery

The registered patients underwent bilateral laparoscopic LLND. The clearance range for LLND included the lymph nodes around the internal iliac artery and the obturator lymph nodes, while the autonomic nerves were generally preserved. Registration was allowed only for cases wherein LLND was performed laparoscopically, regardless of whether TME was performed laparoscopically or robotically.

### Outcome measures

To assess patient safety, the primary outcome was the incidence of Grade III–IV postoperative complications at discharge according to the Clavien–Dindo classification [17]. Proving that the postoperative complication rate in the laparoscopic LLND group is non‐inferior to the rate of postoperative complications in cases of LLND conducted in the JCOG0212 trial would enhance the value of MIS‐LLND [16]. In the JCOG0212 trial, among 351 patients undergoing open LLND, Grade III–IV postoperative complications at discharge occurred in 76 patients. Based on this, the upper limit of the 95% confidence interval was calculated as 27% [16]. For laparoscopic LLND surgery, it was anticipated that the complication rate would decrease by 10%, allowing a 5% margin, with a significance level of 0.1 and power of 0.8. A sample size calculation was performed, and 90 cases was the target number of cases. The secondary outcomes included surgical and pathological outcomes, urinary function, sexual function, disease‐free survival and overall survival.

The criteria for early urinary dysfunction were established based on the definitions used in the JCOG0212 trial [16]. In summary, postoperative urinary dysfunction was assessed by measuring the residual urine volume, typically twice within 5–7 days after surgery. A measurement was considered successful if the total volume of the discharged and residual urine exceeded 150 mL. Dysfunction was defined as having residual urine of ≥50 mL in both measurements. In addition, urinary dysfunction was assessed using the International Prostate Symptom Score (IPSS) [18]. Sexual function was evaluated in male patients using the International Index of Erectile Function (IIEF) [19, 20]. Questionnaire surveys, including the IPSS and IIEF, were administered before and 1 year after surgery.

### Quality control of LLND

**FIGURE 1:**
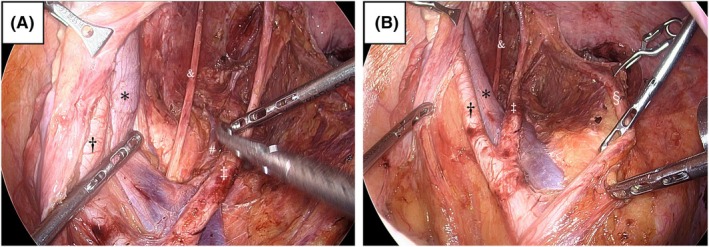
Photographs showing the preservation level of the autonomic nerves and the clearance status of the lymph nodes. (A) Photograph after dissection of the obturator lymph nodes. (B) Photograph after dissection of the lymph nodes around the internal iliac artery. *, the external iliac vein; †, the external iliac artery; ‡, the internal iliac artery; #, the internal iliac vein; &, the obturator nerve; §, the ureter.

Facility requirements for quality control included experience with MIS‐LLND in ≥10 cases or at least 20 lateral pelvic compartments. Surgeon requirements mandated adherence to the endoscopic surgical skill qualification system (ESSQS): a Qualified Surgeon [21]. The ESSQS is a certification system established by the Japan Society for Endoscopic Surgery. It evaluates full‐length videos of applicants performing laparoscopic sigmoidectomy to certify their technical skills as surgeons and instructors. While the number of LLND cases is not a requirement for ESSQS certification, a separate study comparing outcomes of rectal cancer surgeries, including cases with LLND, conducted by ESSQS‐certified and non‐certified surgeons found that surgeries performed by ESSQS‐certified surgeons were associated with less blood loss and fewer postoperative complications [22]. Meetings were held every 3 months at the participating facilities to review the surgical techniques and dissection areas. This study was conducted at six facilities that met the aforementioned conditions. After completion of LLND, two photographs were taken, one on each side, to record the preservation level of the autonomic nerves and the clearance status of the lymph nodes (Figure 1). We assessed whether the inferior hypogastric plexus, pelvic nerves and neurovascular bundles were preserved.

### Statistical analysis

Statistical analyses were performed using the JMP pro 15.1.0 software (SAS Institute, Cary, NC, USA). Non‐inferiority was assessed using a confidence interval approach with a significance level and non‐inferiority margin defined a priori. The results are expressed as the number of cases evaluated for categorical data or as the median and interquartile range (IQR) for quantitative data. Paired *t* tests were used to compare preoperative and postoperative autonomic nervous function assessment scores. Missing data were excluded from the analysis. The study had no dropout cases among the participants.

## RESULTS

### Patient backgrounds and tumour characteristics

Ninety cases were registered between May 2018 and August 2021. Of them, two patients did not undergo protocol treatment and one patient was found to have neuroendocrine carcinoma after registration, leaving 87 cases for the analysis.

Table 1 presents the patient demographics. There were 52 male and 35 female patients with a median age of 66 years and a median body mass index (BMI) of 22.2 kg/m^2^. Thirteen tumour centres were located proximal to the peritoneal reflection, 70 were located between the peritoneal reflection and the upper margin of the anal canal, and four were located within the anal canal. The median distance between the lower edge of the tumour and the anal verge was 5 cm. The lower edge of the tumour was within 3 cm of the anal verge in 29 cases. Regarding baseline stage, there were 30 cases of Stage II and 57 cases of Stage III rectal cancer. The median short‐axis diameter of the lymph node was less than 1 mm. There were no Stage III tumours with lateral lymph nodes >10 mm in the short‐axis diameter. Neoadjuvant chemotherapy was administered in 17 patients.

**TABLE 1:** Patient and tumour characteristics.

Patient characteristics (*N* = 87)	
Sex, no. (%)	
Male	52 (59.8)
Female	35 (40.2)
Age (years), median (IQR)	66 (55, 71)
BMI (kg/m^2^), median (IQR)	22.2 (20.9, 24.7)
Tumour characteristics at initial examination	
Distance from the anal verge (cm), median (IQR)	5.0 (3–6)
The location of the tumour centre, no. (%)	
Proximal to the peritoneal reflection	13 (14.9)
Between the peritoneal reflection and the upper margin of the anal canal	70 (80.5)
Anal canal	4 (4.6)
Tumour stage at baseline, no. (%)	
cT2	2 (2.3)
cT3	76 (87.4)
cT4a	6 (6.9)
cT4b	3 (3.4)
Nodal status at baseline, no. (%)	
cN0	31 (35.6)
cN1	43 (49.4)
cN2	13 (14.9)
Suspected metastasis to lateral lymph node, no. (%)	4 (4.6)
Clinical stage at baseline, no. (%)	
Stage II	30 (34.5)
Stage III	57 (65.5)
Short‐axis diameter of the lateral pelvic lymph node (mm), median (IQR)	0 (0, 4.7)
<5 mm, no. (%)	67 (77.0)
≥5 mm, no. (%)	20 (23.0)
<7 mm, no. (%)	74 (85.1)
≥7 mm, no. (%)	13 (14.9)
Neoadjuvant chemotherapy, no. (%)	
None	70 (80.4)
CAPOX	12 (13.8)
FOLFOX	1 (1.1)
CAPOX + bevacizumab	1 (1.1)
FOLFOX + bevacizumab	1 (1.1)
FOLFOX + panitumumab	1 (1.1)
FOLFOXIRI + bevacizumab	1 (1.1)

Abbreviations: CAPOX, capecitabine and oxaliplatin; BMI, body mass index; FOLFOX, folinic acid, fluorouracil and oxaliplatin; FOLFOXIRI, folinic acid, fluorouracil, oxaliplatin and irinotecan; IQR, interquartile range.

### Surgical outcomes and pathological results

Table 2 displays the surgical outcomes. The median operating time was 472 min, and the median blood loss was 55 mL. Fifty‐five cases underwent TME laparoscopically, while 32 cases were performed robotically. Surgical procedures included anterior resection in 50 cases, intersphincteric resection in four cases and abdominoperineal resection in 32 cases. Overall, 52 of the 54 cases of sphincter‐preserving surgery involved diverting stoma construction. In one case, right‐sided dissection was omitted during LLND. In all cases, the inferior hypogastric plexus, pelvic nerves and neurovascular bundles were preserved. Postoperative complications were observed in 22 cases (25.3%), and the primary outcome of Grade III postoperative complications was observed in five cases (5.7%). This difference is −15.9% compared to the 21.7% complication rate in the JCOG0212 trial, with a 90% confidence interval ranging from −20.9% to −9.5% (*P* < 0.0001). One case developed intestinal obstruction due to stoma outlet obstruction and underwent stoma closure on postoperative day 23. Two cases had anastomotic leakage (2.3%). In an analysis of 20 patients with a BMI of ≥25 kg/m^2^, the median operating time was 528 min (IQR 435–700), the median blood loss was 130 mL (IQR 6–300) and Grade III or higher complications occurred in two (10%) cases, comprising one case of ileus and one case of abdominal infection. Regarding the diverting stoma, closure surgery was performed in 46 of the 52 cases, with a median time to closure of 160 days (IQR 117–224) (median follow‐up period 1471 days; IQR 1172–1839). Among these, one case experienced a rectovaginal fistula after diverting stoma closure. Table 3 shows the pathological results of the surgical specimens. Among the 17 cases receiving neoadjuvant chemotherapy, one achieved complete response pathologically. The median number of lateral lymph nodes harvested bilaterally was eight on each side. Pathological lateral lymph node metastasis was observed in 14 cases, corresponding to a lateral lymph node metastasis rate of 16.1%.

**TABLE 2:** Operative results.

Operative results (*N* = 87)	
Approach used for the TME, no. (%)	
Laparoscopy	55 (63.2)
Robot	32 (36.8)
Surgical procedure, no. (%)	
LAR	50 (57.5)
ISR	4 (4.6)
APR	32 (36.8)
Hartmann	1 (1.0)
Operative duration (min), no. (%)	472 (420, 616)
Blood loss (mL), median (IQR)	55 (20, 175)
Conversion to open surgery, no. (%)	0 (0.0)
Postoperative complications (including duplicates), no. (%)	22 (25.3)
Grade I	4 (4.6)
Grade II	13 (14.9)
Grade III	5 (5.7)
Anastomotic leakage, no. (%)	
Grade II	1 (1.9)^a^
Grade III	1 (1.9)^a^
Ileus, no. (%)	
Grade II	3 (3.4)
Grade III	1 (1.1)
Abdominal infection, no. (%)	
Grade III	3 (3.4)
Urinary retention, no. (%)	
Grade II	6 (6.9)
Others, no. (%)	
Grade I	4 (4.6)
Grade II	5 (5.7)
Grade III	1 (1.1)
Reoperation within 30 days post‐surgery, no. (%)	1 (1.1)
30‐day mortality, no. (%)	0 (0.0)

Abbreviations: APR, abdominoperineal resection; IQR, interquartile range; ISR, intersphincteric resection; LAR, low anterior resection; TME, total mesorectal excision.

^a^ The percentage of the 54 cases in which an anastomosis‐preserving sphincter surgery was performed.

**TABLE 3:** Pathological results.

Pathological results	
Tumour diameter (mm), median (IQR)	50 (40, 60)
Histological type (excluding one pCR case), no. (%)	
Tubular adenocarcinoma	79 (91.9%)
Mucinous adenocarcinoma	6 (7.0%)
Papillary adenocarcinoma	1 (1.2%)
pT, no. (%)	
pT0	1 (1.1%)
pTis	2 (2.3%)
pT1	1 (1.1%)
pT2	24 (27.6%)
pT3	53 (60.9%)
pT4a	2 (2.3%)
pT4b	4 (4.6%)
pN, no. (%)^†^	
N0	44 (50.6%)
N1	22 (25.3%)
N2	21 (24.1%)
Number of lateral pelvic lymph nodes, median (IQR)	
Right	8 (6, 11)
Left	8 (5, 11)
Number of cases with metastasis to the lateral lymph node, no. (%)	14 (16.1%)
Proximal resection margin, no. (%)	
Negative	87 (100%)
Positive	0 (0%)
Distal resection margin, no. (%)	
Negative	87 (100%)
Positive	0 (0%)
Radial margin, no. (%)	
Negative	85 (97.7%)
Positive	2 (2.3%)

Abbreviations: CR, complete response; IQR, interquartile range.

### Autonomic nervous function assessment

The results of the IPSS and IIEF surveys conducted to assess the preservation of autonomic nervous function are shown in Table 4. The response rates for the questionnaires were >70% in the preoperative assessment and >60% in the assessment at 1 year postoperatively. There were no significant differences in the urinary or sexual function between preoperative and 1‐year postoperative comparisons. Regarding the evaluation of urinary dysfunction using residual urine measurements, the results showed that, of the 73 participants, 24 had urinary dysfunction. Including one case of urinary retention, wherein residual urine measurement could not be conducted, the incidence rate of urinary dysfunction was 34%.

**TABLE 4:** Autonomic nervous function assessment.

	Before surgery	1 year after surgery	*P* value	Response rate for the questionnaires (before surgery/1 year after surgery)
IPSS				
Symptom score total	4.5 (2, 8)	5 (2.25, 10)	0.1682	83%/61%
Quality of life	2 (1, 3)	2 (1, 3)	0.3733	82%/72%
IIEF^a^				
Erectile function	3 (1, 23)	4 (1.25, 11.75)	0.0938	75%/62%
Orgasmic function	0 (0, 8)	0.5 (0, 6.5)	0.2066	75%/73%
Sexual desire	4 (2, 6)	4 (2, 5)	0.8104	75%/69%
Intercourse satisfaction	0 (0, 4)	0 (0, 3)	0.4494	75%/73%
Overall satisfaction	6 (6, 8)	6 (6, 7.75)	0.6784	75%/62%

Abbreviations: IIEF, International Index of Erectile Function; IPSS, International Prostate Symptom Score.

^a^ Evaluated only for male patients.

## DISCUSSION

In this study, we demonstrated that prophylactic laparoscopic LLND can be safely performed under stringent quality control. Previous studies on laparoscopic LLND have primarily been retrospective, with limited prospective studies. Although the oncological significance of LLND has been demonstrated in trials, such as the JCOG0212 trial, its high invasiveness prevents its widespread adoption. By establishing the safety of laparoscopic LLND in this study, indications for LLND can be reconsidered.

In the JCOG0212 trial, the non‐inferiority of TME with LLND to TME alone was not proven in terms of the primary endpoint of relapse‐free survival, which indicated that TME with LLND is the standard treatment [6]. However, given the increasing prominence of MIS in modern treatment, extrapolating LLND indications from the JCOG0212 trial, which focused exclusively on open surgery, has become a challenge. Therefore, considering the importance of adequate quality control, similar to the JCOG0212 trial, we deemed it necessary to evaluate laparoscopic LLND. Safety was prioritized as the primary endpoint, and the results demonstrated that laparoscopic LLND could be safely performed. Furthermore, the complication rate of 5.7% in this study was much lower than the initial hypothesis and notably lower than the 22% complication rate reported in the TME with LLND group of the JCOG0212 trial. In clinical trials of laparoscopic surgery for rectal cancer conducted to date, the incidence rate of Grade III or higher complications has been reported to be approximately 10%–20%. Considering this, it can be concluded that the safety of the surgery was well maintained in this trial [23, 24, 25, 26, 27]. The longer operating time and lower blood loss observed in our study compared with open surgery are consistent with the characteristics of laparoscopic surgery compared with open surgery, and are within the acceptable range [28]. Recognizing the differences in surgical procedures performed when comparing the postoperative complication rates between JCOG0212 and this study is necessary. The proportion of sphincter‐preserving surgery and abdominoperineal resection was 81%/19% in the LLND group of JCOG0212, compared to 62%/37% in this study, suggesting that the cohort in this study included more cases with tumours located closer to the anus. Therefore, it cannot be ruled out that the lower incidence of anastomotic leakage and the favourable rates of Grade III or higher postoperative complications observed in this study were influenced by this difference in surgical procedure.

Since the inclusion of robotic surgery in the insurance coverage in Japan in 2018, the proportion of rectal cancer surgeries performed robotically for LLND has been increasing [29]. Although this study was planned for 2017, which was before robot‐assisted LLND became widespread, the delicate operability of robotic surgery is still considered beneficial for LLND. In the VITRUVIANO trial, which included approximately 23% of patients undergoing LLND, the Grade III or higher complication rate was 6.3% [30]. Considering this, the 5.7% Grade III complication rate in our study, which focused solely on LLND cases, can be considered favourable.

In this study, LLND that preserved the autonomic nerves was performed. While the autonomic nerves were preserved in all cases, urinary dysfunction occurred in 34% of the patients based on postoperative residual urine measurements. The evaluation method of residual urine used in this study was as stringent as the methods adopted in other studies, such as in the JCOG0212 trial, and the resulting incidence of urinary dysfunction was considered acceptable compared to those studies [31]. Moreover, based on the IPSS at 1 year postoperatively, long‐term urinary function was considered good. Similarly, regarding the evaluation of male sexual function based on the IIEF, it was confirmed that male sexual function had good recovery 1 year postoperatively. These results suggest that adequate quality‐controlled laparoscopic LLND is feasible from a functional preservation perspective.

The lateral pelvic lymph node metastasis rate in this study was 16.1%, which was higher than the 7% reported in the JCOG0212 trial. To meet the eligibility criteria of this study, the short axis of the lateral pelvic lymph nodes needed to be <10 mm, which is consistent with the JCOG0212 trial. However, upon closer examination of the JCOG0212 trial, 13% of the cases had lymph nodes measuring ≥5 mm, while our study had lymph nodes measuring ≥5 mm in 23% of the cases [32]. Therefore, it is reasonable to conclude that the pathological lymph node metastasis rate was higher. In this study, some cases underwent neoadjuvant chemotherapy, which might have led to the disappearance of micrometastases in the lateral lymph nodes. However, since JCOG0212 included only surgery‐first cases, it cannot be ruled out that the positive rate of lateral lymph node metastasis at diagnosis differed even more significantly between JCOG0212 and this study. According to Akiyoshi et al., in predicting pathological lateral lymph node metastasis, the cutoff value for lymph node size was a short axis of 8 mm, with a metastasis rate of 20% for nodes measuring <8 mm [33]. The median lymph node size in this study was less than 1 mm, suggesting that the lymph node size in this study was small compared to this criterion. In our previous study, considering the results of positron emission tomography along with the cutoff value of a 7 mm short axis was effective for detecting pathological metastasis [34]. In the future, both the surgical technique and the indications for LLND will also be important issues.

Our group has previously conducted prospective clinical studies to develop neoadjuvant chemotherapy for advanced rectal cancer [35, 36, 37]. In Japan, local control equivalent to that achieved in Western countries was obtained through rectal resection with LLND, which had been the standard treatment. This led to the introduction of neoadjuvant chemotherapy to control not only the primary lesion but also systemic cancer progression while avoiding the adverse effects associated with radiation therapy. Consequently, neoadjuvant chemotherapy for advanced rectal cancer was established as one of the standard treatments within our group. However, performing LLND under the tissue changes caused by radiation therapy, including oedema and fibrosis, was considered to carry additional risks. Therefore, in this clinical trial, which aimed to evaluate the safety of the procedure, cases involving prior radiation therapy were excluded. Currently, favourable outcomes from neoadjuvant chemotherapy have been reported in major global clinical trials [38, 39]. This study, which demonstrated the safety of LLND even in cases involving neoadjuvant chemotherapy, is considered significant.

Patients in Western countries generally have a higher BMI than those in Japan. Additionally, preoperative treatments, including radiation therapy such as chemoradiotherapy or total neoadjuvant therapy, are commonly performed, with LLND not being a standard procedure. However, recent reports have indicated that, even in cases where such preoperative treatments are administered, performing LLND for patients with enlarged lateral lymph nodes can achieve favourable local control [10, 40]. In the subgroup analysis of this study, obesity did not compromise surgical safety, with outcomes comparable to those reported in JCOG0212 and within acceptable ranges. Considering these findings, even in Western countries where preoperative therapy is the standard, LLND may hold significant value in certain cases. In the current era of widespread minimally invasive treatments, research to validate the safe implementation of LLND is crucial. However, when radiation therapy is administered preoperatively, there is an inherent risk of increased surgical difficulty. Therefore, caution is essential when introducing LLND in such settings.

This study has several limitations. In Japan, lymph node evaluation is performed by removing the mesorectum, resulting in a lack of data on circumferential resection margins, which are surrogate markers for long‐term prognosis [41]. A long‐term outcome analysis has not yet been conducted, and the evaluation of oncological validity is incomplete; hence, a long‐term outcome analysis should be conducted in the future. In addition, as a single‐arm study without a comparative design, direct comparison with the JCOG0212 trial, which occurred in a different era, is challenging.

In conclusion, we demonstrated the safety of prophylactic laparoscopic LLND. The data highlight the minimally invasive nature of laparoscopic surgery for LLND and provide valuable data for considering the indications of LLND in MIS.

## AUTHOR CONTRIBUTIONS

**Atsushi Hamabe:** Conceptualization; project administration; data curation; investigation; formal analysis; methodology; validation; writing – original draft. **Junichi Nishimura:** Conceptualization; project administration; data curation; investigation; formal analysis; methodology; validation; visualization; writing – original draft. **Yozo Suzuki:** Investigation; data curation; formal analysis; methodology; validation. **Masayoshi Yasui:** Investigation; data curation; methodology; validation. **Masakazu Ikenaga:** Investigation; data curation; methodology; validation. **Tsukasa Tanida:** Investigation; data curation; methodology; validation. **Shinichi Yoshioka:** Investigation; data curation; methodology; validation. **Yoshihito Ide:** Investigation; data curation; methodology; validation. **Yusuke Takahashi:** Investigation; data curation; methodology; validation. **Hiroshi Takeyama:** Investigation; data curation; methodology; validation. **Takayuki Ogino:** Investigation; data curation; methodology; validation. **Hidekazu Takahashi:** Investigation; data curation; methodology; validation. **Norikatsu Miyoshi:** Investigation; data curation; methodology; validation; writing – review and editing. **Makoto Fujii:** Formal analysis; methodology; validation. **Yuko Ohno:** Formal analysis; methodology; validation. **Hirofumi Yamamoto:** Conceptualization; project administration; supervision; writing – review and editing. **Kohei Murata:** Conceptualization; project administration; supervision; writing – review and editing. **Mamoru Uemura:** Conceptualization; project administration; investigation; data curation; supervision; methodology; validation; visualization; writing – review and editing. **Yuichiro Doki:** Conceptualization; project administration; supervision; writing – review and editing. **Hidetoshi Eguchi:** Conceptualization; project administration; supervision; writing – review and editing.

## FUNDING INFORMATION

The study did not receive any form of funding.

## CONFLICT OF INTEREST STATEMENT

The authors declare no conflict of interests for this article.

## ETHICS STATEMENT

The ethics committee of each participating institution approved the study protocol. All informed consent was obtained from the participants. This study was registered with the University Hospital Medical Information Network Clinical Trials Registry (identification number UMIN000033355).

## Data Availability

The data that support the findings of this study are available on request from the corresponding author. The data are not publicly available due to privacy or ethical restrictions.
